# Assessment of Glycometabolism Impairment and Glucose Variability Using Flash Glucose Monitoring System in Patients With Adrenal Diseases

**DOI:** 10.3389/fendo.2020.544752

**Published:** 2020-09-25

**Authors:** Minmin Han, Xiaoming Cao, Changjian Zhao, Luyang Yang, Nan Yin, Pengliang Shen, Jin Zhang, Fei Gao, Yi Ren, Dong Liang, Jing Yang, Yi Zhang, Yunfeng Liu

**Affiliations:** ^1^First Clinical Medical College, Shanxi Medical University, Taiyuan, China; ^2^Department of Endocrinology, First Hospital of Shanxi Medical University, Taiyuan, China; ^3^Department of Urology, First Hospital of Shanxi Medical University, Taiyuan, China; ^4^Department of Pharmacology, Shanxi Medical University, Taiyuan, China

**Keywords:** glucose metabolism impairment, flash glucose monitoring system, Cushing syndrome, primary aldosteronism, pheochromocytoma, nonfunctional adrenal incidentaloma, glucose variability

## Abstract

**Background:**

This study aimed to investigate the characteristics and extent of glycometabolism impairment in patients with adrenal diseases, including Cushing syndrome, primary aldosteronism, pheochromocytoma, and nonfunctional adrenal incidentaloma.

**Methods:**

This study enrolled thirty-two patients with adrenal diseases as adrenal disease groups and eight healthy individuals as healthy controls. Blood glucose levels were indicated by glucose concentration in interstitial fluid, which was documented using flash glucose monitoring system. According to flash glucose monitoring system data, parameters representing general blood glucose alterations, within-day and day-to-day glucose variability, and glucose-target-rate were calculated. Furthermore, blood glucose levels at nocturnal, fasting, and postprandial periods were analyzed. Besides, islet β-cell function and insulin resistance were assessed.

**Results:**

Analysis of flash glucose monitoring system-related parameters indicated impaired glycometabolism in patients with adrenal diseases compared with that of healthy controls at general blood glucose, within-day and day-to-day glucose variability, and glucose-target-rate levels. Furthermore, the dynamic glucose monitoring data revealed that significantly affected blood glucose levels compared with that of healthy controls were observed at postprandial periods in the Cushing syndrome and primary aldosteronism groups; at nocturnal, fasting and postprandial periods in the pheochromocytoma group. Significant insulin resistance and abnormal β-cell function were observed in the Cushing syndrome group compared with that in healthy controls.

**Conclusion:**

Adrenal diseases can negatively affect glucose metabolism. Patients diagnosed with adrenal diseases should receive timely and appropriate treatment to avoid adverse cardiovascular events linked to hyperglycemia and insulin resistance.

## Introduction

Adrenal glands are critical endocrine glands in the human body and responsible for various physiological functions such as metabolism of glucose, protein, lipid, water, and saline. Primary mechanisms underlying these functions are dependent on hormones secreted by the adrenals, mainly comprising glucocorticoids (GCs), aldosterone (ALD), and catecholamine (CA). Adrenal diseases, including Cushing syndrome (CS), primary aldosteronism (PA), and pheochromocytoma (Pheo) are linked to varying degrees of metabolic abnormalities by excessively secreted hormones. Impaired glucose metabolism may be one of the reflections of abnormal adrenal function. Diabetes mellitus secondary to CS, PA, and Pheo has typically been classified as a specific type of diabetes mellitus by the American Diabetes Association (1). Recent data reveal that patients with nonfunctional adrenal incidentaloma (NFAI) may experience undetectable autonomous cortisol secretion and exhibit a higher incidence of impaired glucose tolerance and insulin resistance (IR) than controls (2, 3).

The mechanisms underpinning adrenal diseases posing effect on glucose metabolism are multifactorial (4–6). To explore this issue, glucose metabolism (uptake, disposal, and storage) in major glycometabolic organs (liver, skeletal muscle, and adipose tissue) and islet β-cell function need to be examined. In conditions with excessive adrenal hormones, gluconeogenesis in liver is stimulated and glucose uptake in skeletal muscle is reduced. Impaired insulin secretion and insulin resistance are known to be involved in this process.

The influences of adrenal diseases, including CS, PA, Pheo, and NFAI on glucose metabolism are receiving increasing interest because of their escalating prevalence in the general population. Worth noting is that hyperglycemia and IR are significantly correlated with adverse cardiovascular events which contribute to the primary cause of death in these patients (7, 8). Therefore, the relationship between glucose metabolism and adrenal diseases is now an area of growing interest.

Convincing evidence in the literature has demonstrated that patients with CS, PA, Pheo, and NFAI are inclined to experience impaired glucose metabolism, but few attempts have been made to elucidate the glycometabolism of these patients in terms of dynamic blood glucose surveillance. The aim of this study was therefore to investigate the extent and patter of glucose metabolism impairment in patients with CS, PA, Pheo, and NFAI using flash glucose monitoring system (FGMS, FreeStyle Libre, Abbott Laboratories).

Although there have been multiple lines of experimental and clinical researches shedding light on glycometabolism impairment in patients with CS, PA, Pheo, and NFAI, our study is distinct in that we employed FGMS to document consistent blood glucose levels during the day. FGMS records interstitial glucose values, which are corroborated to reflect plasma glucose concentration accurately after a short time lag, every 15 min daily. A 24-h surveillance provides 96 blood glucose values.

In comparison with the traditional finger-prick blood glucose monitoring regimen, FGMS documents consecutive interstitial glucose concentrations, which can commendably reflect blood glucose fluctuations during the day and exhibit elaborate ambulatory glucose profile, providing useful information about general blood glucose alterations, glucose variability (GV) characteristics, and glucose-success-rate. The usage of FGMS in this study can uncover the extent and pattern of glycometabolism impairment in patients with adrenal diseases, combining with assessment of β-cell function and IR, further elucidating mechanisms underlying these metabolic changes and offering recommendations for blood glucose management in these patients.

## Materials and Methods

### Ethics and Setting

This clinical study was conducted in the Departments of Endocrinology and Urology in the First Hospital of Shanxi Medical University from November 2018 to October 2019. This study was approved by the ethics committee of the First Hospital of Shanxi Medical University (2018K006). Written informed consent was obtained from all recruited subjects.

### Subjects

Patients who were admitted to the Departments of Endocrinology and Urology in the First Hospital of Shanxi Medical University from November 2018 to October 2019 and diagnosed with adrenal diseases were included in this study. Of these patients, five diagnosed with CS, nine with PA, ten with Pheo, and eight with NFAI were qualified to be included in adrenal disease groups (ADs). Eight healthy volunteers were recruited as healthy controls (HCs). HCs declared no history of hypertension, diabetes mellitus, obesity, or electrolyte disturbance. The ADs and HCs did not take medications that affect blood glucose levels, such as hypoglycemic drugs and dexamethasone etc. during the study period. Each ADs and HCs matched for gender, age, and body mass index. Endocrine assessment was conducted 24 h after admission.

Patients included in the CS group had to conform to the following criteria (9): typical symptoms and signs (centripetal obesity, moon face, hirsutism, red-purple striae etc.); adrenal adenoma proved by adrenal computed tomography (CT); lower than normal adrenocorticotropic hormone (<1.6 nmol/L) with concurrently aberrant cortisol rhythm (midnight cortisol>200nmol/L); increased 24-h urinary free cortisol (>379 nmol/L); unsuppressed cortisol level after overnight 1-mg and 8-mg dexamethasone suppression test (DST); postoperative pathology report indicating adrenocortical adenoma and elimination of other potential factors (chronic anxiety, psychotropic medications, exogenous GCs exposure, stress from venipuncture, etc.) which may influence endogenous cortisol levels.

Patients qualified for inclusion in the PA group had to meet the following standards (10): typical symptoms of hypokalemia and hypertension; adrenal adenoma identified by adrenal CT; increased ALD level and suppressed plasma renin activity (PRA); ALD (pg/L) to PRA (ng/ml/h) ratio (ARR)>300; postoperative pathology report indicating adrenocortical adenoma and restored plasma potassium after surgery; elimination of potential factors (antihypertension drugs, glycyrrhiza extracts, etc.) which may affect ARR measurement. Patients who were taking β-blockers, angiotensin-converting-enzyme inhibitors, angiotensin receptor blockers and diuretics underwent a 2-week drug washout before ARR assessment and were advised to take amlodipine as antihypertensive treatment.

Patients were included in the Pheo group according to sporadic hypertension accompanied with sustained hypertension, and palpitations; adrenal CT findings; postoperative histopathology reports. Plasma and urinary CA as well as their metabolites were measured.

The NFAI group displayed adrenal adenoma on CT scan and normal hormone secretion identified by endocrine profile.

### FGMS

FGMS is composed of a sensor and scanning reader. The sensor is installed on the lateral arm of the subject. Blood glucose levels were intended to be monitored by FGMS in ADs and HCs for 14 days. However, complete 14-day glucose monitoring was not obtained due to accidental dropping of the sensor, or the patients required CT, nuclear magnetic resonance imaging, or surgery during the surveillance period. The FGMS recorded glucose values every 15 min under conditions of a real-time glucose value scanned by the reader within 8 h. Several time points were missed because of poor subject compliance and other unavoidable factors, such as sleep time exceeding 8 h. Missing values were added with the mean of values before and after the missing values. Due to the poor accuracy (11), the FGMS data at the first day of installation were excluded, In the CS group, 45-day glucose values were obtained (one patient, 5 days; one patient, 7 days; two patients, 10 days; and one patient, 13 days). The PA group provided 68-day glucose values (four patients, 4 days; one patient, 5 days; one patient, 9 days; one patient, 12 days; and two patients, 13 days). The Pheo group provided 74-day glucose values (one patient, 4 days; three patients, 6 days; four patients, 7 days; one patient, 11 days; and one patient, 13 days). The NFAI group provided 85-day glucose values (two patients, 7 days; one patient, 8 days; two patients, 12 days; and three patients, 13 days). In HCs, 54-day glucose monitoring was accomplished (7 patients, 7 days; and one patient, 5 days). All the subjects were recommended regular meals at 08:00, 13:00, and 18:00 over the course of surveillance. They were instructed to maintain similar levels of physical activity, and received same calorie intake (25kcal/kg/day) during the monitoring period. According to the latest Dietary Guideline for Chinese Residents, the total calorie intake distributed to the three meals in a proportion of 3:4:3, including 60% carbohydrate, 15% protein, and 25% fat.

### Laboratory Analysis

Glycosylated hemoglobin (HbA1c, %) was measured to gauge the mean blood glucose level of most recent 2–3 months. All the recruited subjects received tests for fasting blood glucose (FBG, (mmol/L) and fast insulin (FINS, µIU/ml). IR and islet β-cell function were assessed by homeostasis model assessment-IR (HOMA-IR), quantitative insulin sensitivity check index (QUICKI), fasting glucose to insulin ratio (G/I), and HOMA-β,

### Data Analysis

SPSS 19.0 and SigmaPlot 12.5 were used for data analysis and graph construction. P<0.05 was considered statistically significant. All values are denoted as mean ± standard deviation unless otherwise stated. Some data are represented as median (first quartile, third quartile) due to the wide distribution.

IR and islet β-cell function were assessed using the following formulas (12–14): HOMA-IR=(FBG×FINS)/22.5; QUICKI=1/[(logFINS)+(logFBG)]; G/I= FBG/FINS; HOMA-β=(20×FINS)/(FBG-3.5). These indices and HbA1c, FBG, and FINS were compared between ADs and HCs by compared two-group t-test (for normally distributed data) or Mann-Whitney test (for skewed data).

FGMS data were disposed from two perspectives. First of all, parameters representing general blood glucose alterations, within-day GV, day-to-day GV, and glucose-target-rate were calculated according to FGMS monitoring data (15–19). Secondly, several time periods were analyzed specifically, including nocturnal, fasting, and postprandial periods. According to HbA1c data, one could speculate that subjects in ADs did not present substantial blood glucose elevation. Therefore, the normal range of glucose values was set as 3.9 mmol/L to 7.8 mmol/L for proper and detailed identification of increased blood glucose in ADs.

FGMS-related parameters were listed in the **Table 1** and compared between four ADs and HCs by a t-test or Mann-Whitney test. Five time periods were extracted for specific disposal: 00:00–06:00 (nocturnal period), 06:00–08:00 (fasting period), and 08:00–10:00, 13:00–15:00, 18:00–20:00 (postprandial periods). In the CS group, the mean glucose value at each time point of each subject was drawn as multiple-line and scatter diagram and the area under the curve (AUC) of each analyzed time period was calculated. In the same manner, AUCs for the other ADs and HCs were computed. A t-test or Mann-Whitney test was used to compare the AUC of each time period between four ADs and HCs, respectively.

**Table 1:** Flash glucose monitoring system (FGMS)-related parameters.

Types	Parameters	Representations
General blood glucose alterations	24hMBG	the mean value of 96 blood glucose values during the day
	CV	standard deviation/MBG
within-day GV	SDBG	the standard deviation of 96 blood glucose values during the day
	MAGE	the mean value of glucose excursion amplitudes above 1 SDBG, whose directions are in line with the first appeared glucose excursion amplitude above 1 SDBG
Day-to-day GV	MODD	the mean value of absolute values of the blood glucose values difference at the same time point in two adjacent days
	ABPC	The 25^th^ and 75^th^ percentiles of each time point for each subject during the glucose surveillance period were calculated and drawn as double-line and scatter diagram. Then, the ABPC was calculated.
glucose-target-rate	PT1	the percentage of blood glucose values less than 3.9mmol/L during the day
	PT2	the percentage of blood glucose values within the range of 3.9–7.8mmol/L during the day
	PT3	the percentage of blood glucose values above 7.8mmol/L during the day
	TIR	the time of blood glucose values within 3.9–7.8 mmol/L during the day
	TOR	the time of blood glucose values below and above TIR during the day

GV, glucose variability; SD, standard deviation; 24hMBG, 24-h mean blood glucose; CV, coefficient of variance; SDBG, SD of blood glucose; MAGE, mean amplitude of glycemic excursions; MODD, mean of daily differences; ABPC, area between the 25th and 75th percentile curves; PT, percentile time; TIR, time in range; TOR, time out of range.

## Results

The characteristics of the recruited subjects were showed in **Supplementary File**, including gender, age, body mass index, blood pressure, and plasma potassium for all the subjects; disease duration, tumor size, and pathological result for ADs; and endocrinological data listed in the inclusion criteria for each ADs. The disease duration was determined in two perspectives: based on tumor size and history taking. Due to the subtle onset and time-dependent progression, we cannot determine the real disease duration of the subjects in ADs. However, recent data show that benign adrenal tumors and Pheo grow through a lengthy process at a rate less than 0.3 cm/year (20, 21), therefore, the disease duration was estimated according to the tumor size and its growth rate. On the other hand, the disease duration based on history taking was defined by following criteria; duration of typical Cushing manifestations (centripetal obesity, moon face, or purple striae etc.), or hypertension in the CS group; duration of hypertension, or hypokalemia in the PA group; duration of hypertension in the Pheo group; and duration of hypertension, or adrenal incidentaloma in NFAI group.

**Figure 1:**
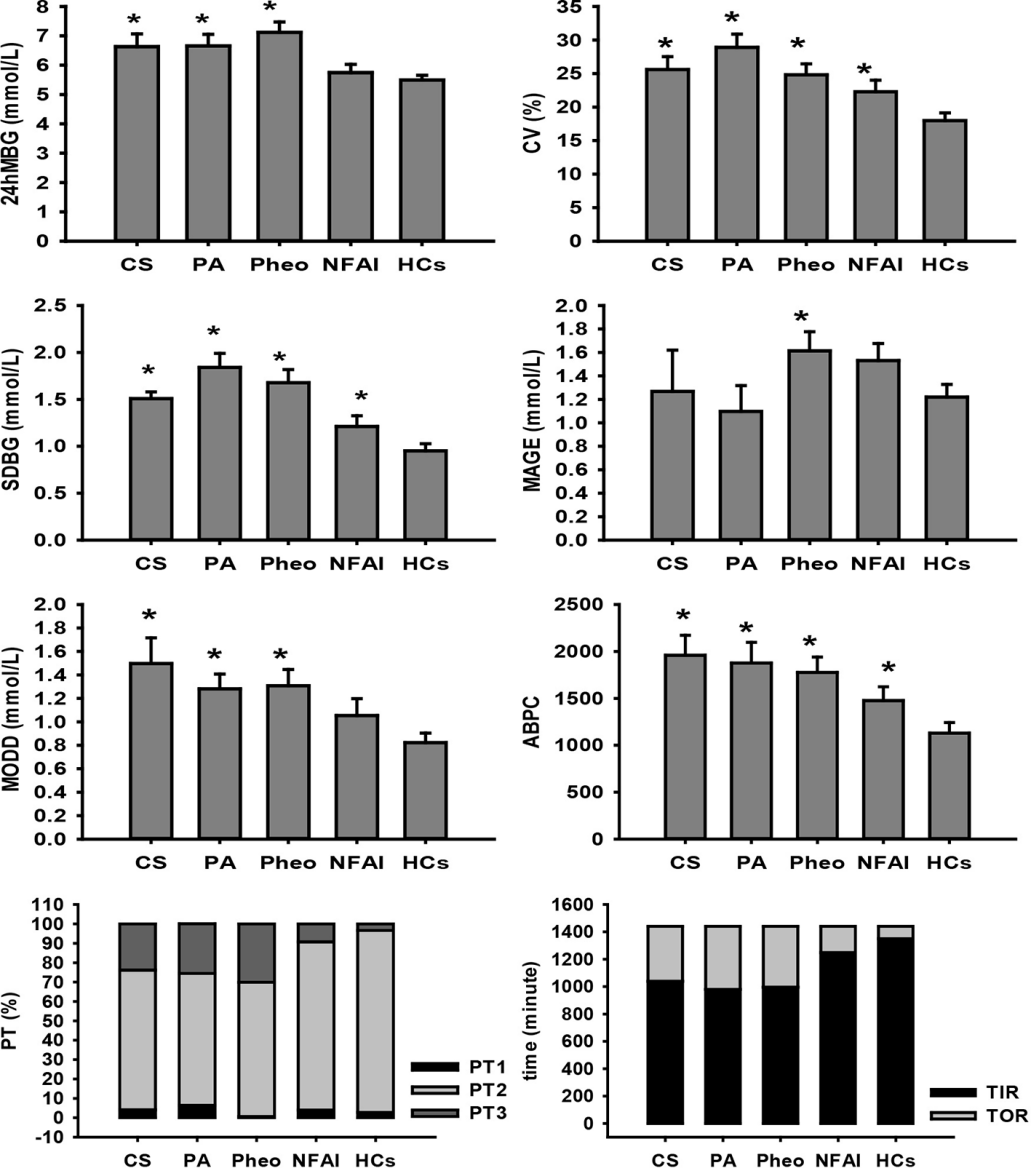
Comparison of FGMS-related parameters in ADs and HCs. * represents significant difference between ADs and HCs. The data were showed as mean ± SEM. FGMS, flash glucose monitoring system; ADs, adrenal disease group; HCs, healthy controls; SEM, standard error of mean.

In the CS group, all five recruited subjects had typical Cushing symptoms, adrenocortical adenoma by postoperative pathological report, deranged cortisol rhythm, and unaffected cortisol concentration after overnight 1-mg and 8-mg DST; the average PRA upright was 2.26 (1.97, 5.43) ng/ml/h (0.93–6.56 ng/ml/h); the average ALD upright was 115.92 ± 34.82 pg/ml (65–296 pg/ml); the mean disease duration based on tumor size and history taking was 9.6 ± 2.0 years and 4.7 ± 1.92 years, respectively. Significantly different 24hMBG (P=0.003) and increased CV (P=0.002) were identified when compared with those of HCs. Parameters representing within-day and day-to-day GV showed that SDBG was significantly higher (one-tailed t-test; P=0.001), MODD and ABPC was significantly different (P=0.006, and P=0.006, respectively) when compared with those of HCs, while MAGE didn’t find any significant difference from that in HCs. Indices indicating glucose-success-rate were found significant differences in PT2, PT3, TIR, and TOR compared with those of HCs (P=0.002, P=0.011, P=0.002, and P=0.002, respectively), indicating that higher TOR in the CS group than in HCs was mainly composed of PT3 (**Figure 1**). As for other calculated indices, HOMA-IR and HOMA-β were higher, and QUICKI was lower than that in HCs (one-tailed t-test; P=0.031, P=0.024, and P=0.034, respectively); G/I and FINS differed significantly from that in HCs (P=0.03, and P=0.015, respectively); no significant difference was observed in HbA1c and FBG (**Table 2**).

**Table 2:** Metabolic parameters in ADs and HCs.

Parameters	CS	PA	Pheo	NFAI	HCs
HbA1c(%)	6.2 ± 1.48	6.2 ± 0.62*	6.6 ± 0.70*	5.4 ± 0.54	5.3 ± 0.41
FBG(mmol/L)	5.5 ± 1.13	5.4 ± 0.79	6.2 ± 1.25*	5.4 ± 1.14	5.3 ± 0.62
FINS(μU/ml)	11.29(10.92, 11.3)*	7.1(5.4, 7.3)	5.9(3.5, 7.2)	8.3(4.5, 9.5)	5.4(4.9, 8.2)
HOMA-IR	2.7 ± 0.97*	1.7 ± 1.02	2.0 ± 1.71	1.8 ± 0.89	1.6 ± 0.85
HOMA-β	157.8(87.39, 162.99)*	71.3 (41.9, 97.1)	45.6(35, 61.2)	96.5 (61.0, 125.1)	77.52(47.4, 110.02)
QUICKI	0.6 ± 0.06*	0.7 ± 0.15	0.7 ± 0.12	0.7 ± 0.16	0.7 ± 0.10
G/I	0.5 ± 0.09*	1.1 ± 0.77	1.2 ± 0.50	1.0 ± 0.74	0.9 ± 0.32

ADs, adrenal disease groups; HCs, healthy controls; CS, Cushing syndrome; PA, primary aldosteronism; Pheo, pheochromocytoma; NFAI, nonfonctional adrenal incidentaloma; HbA1c, Glycosylated hemoglobin; FBG, fasting blood glucose; FINS, fasting insulin; HOMA-IR, homeostasis assessment-insulin resistance; QUICKI, quantitative insulin sensitivity check index; G/I, fasting glucose to insulin ration. * represents significant difference between adrenal disease groups and HCs.

In the PA group, all recruited subjects exhibited hypokalemia, hypertension, adrenocortical adenoma by postoperative pathological report, decreased PRA, and ARR>300; the mean plasma potassium was 2.939 ± 0.333 mmol/L (3.5–4.5 mmol/L); the average blood pressure was 174/100 ± 11/13 mmHg; the cortisol rhythm was normal and mean cortisol at 08:00 was 315.82 ± 117.32 nmol/L (171–536 nmol/L); the average adrenocorticotropic hormone level was 4.22 ± 2.85 pmol/L (1.6–13.9 pmol/L); the mean disease duration based on tumor size and history taking was 6.07 ± 2.51 years and 3.94 ± 1.84 years, respectively. 24hMBG was significantly different from (P=0.024) and CV was significantly higher (one-tailed t-test; P<0.001) than that of HCs. Parameters representing within-day and day-to-day GV showed that SDBG and MODD were significantly higher (one-tailed t-test; P<0.001, and P=0.005, respectively) and ABPC was significantly different (P=0.024) compared with those of HCs, while MAGE didn’t find any significant difference from that in HCs. All indices indicating glucose-target-rate were found significant differences compared with those of HCs except PT1, suggesting that PT3 was the main part of the increased TOR in the PA group (**Figure 1**). General HbA1c level exceeded that of HCs (P=0.001), whereas, FBG and FINS along with indices of IR and islet β-cell function have no significant differences from those of HCs (**Table 2**).

In the Pheo group, all subjects diagnosed with Pheo were confirmed by adrenal CT findings and postoperative pathological reports; the average blood pressure of the patients in this group was 160/99 ± 24/14 mmHg; the average PRA upright was 4.45 ± 1.80 ng/ml/h (0.93–6.56 ng/ml/h); the average ALD upright was measured as 152.06 ± 56.82 pg/ml (65–296 pg/ml); all of the Pheo patients presented normal cortisol rhythm and content; the mean disease duration based on tumor size and history taking was 16.02 ± 6.41 years and 5.85 ± 2.16 years, respectively. Significantly increased 24hMBG and CV were identified than that in HCs by one-tailed t-test (P<0.001, and P=0.003, respectively). Indices of within-day GV were significantly higher than those of HCs (one-tailed t-test; SDBG: P<0.001, and MAGE: P=0.038). Significantly different MODD (P=0.006) and increased ABPC (P=0.004) was identified compared with those of HCs. All indices indicating glucose-target-rate were found significant differences compared with those of HCs except PT1, indicating that the higher TOR in the Pheo group than in HCs was mainly composed of PT3 (**Figure 1**). A significant difference with that in HCs was found in HbA1c (P<0.001) and FBG was significantly higher than that in HCs (one-tailed t-test; P=0.044), while no significant differences were reported in FINS, and parameters of IR and islet β-cell function (**Table 2**).

In the NFAI group, all subjects exhibited no hormone over-secretion and adrenal adenoma in adrenal CT; the mean disease duration based on tumor size and history taking was 7.39 ± 3 years and 25 (13, 112.5) days, respectively. Significantly higher CV and TOR along with significantly lower PT2 and TIR were observed when compared with those of HCs (P=0.029, P=0.025, P=0.025, and P=0.025, respectively; **Figure 1**). HbA1c, FBG, and FINS along with parameters representing IR and islet β-cell function showed no significant difference compared with those of HCs (**Table 2**).

**Figure 2:**
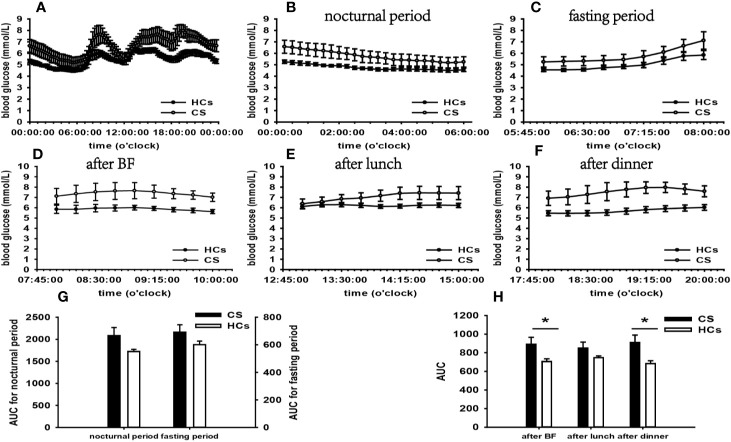
Comparison of the mean blood glucose level and AUC between the CS group and HCs. Mean blood glucose level: **(A)** at each time point during the day; **(B)** at nocturnal period; **(C)** at fasting period; **(D)** after BF; **(E)** after lunch; **(F)** after dinner. AUC: **(G)** at nocturnal and fasting periods; **(H)** at postprandial periods. * represents significant difference between CS and HCs. The data were showed as mean ± SEM. AUC, area under the curve; CS, Cushing syndrome; HCs, healthy controls; BF, breakfast; SEM, standard error of mean.

**Figure 3:**
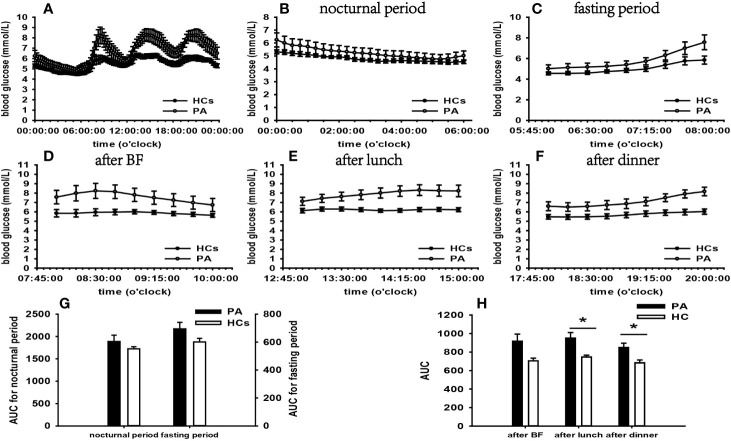
Comparison of the mean blood glucose level and AUC between the PA group and HCs. Mean blood glucose level: **(A)** at each time point during the day; **(B)** at nocturnal period; **(C)** at fasting period; **(D)** after BF; **(E)** after lunch; **(F)** after dinner. AUC: **(G)** at nocturnal and fasting periods; **(H)** at postprandial periods. * represents significant difference between PA and HCs. The data showed as mean ± SEM. AUC, area under the curve; PA, primary aldosteronism; HCs, healthy controls; BF, breakfast; SEM, standard error of mean.

**Figure 4:**
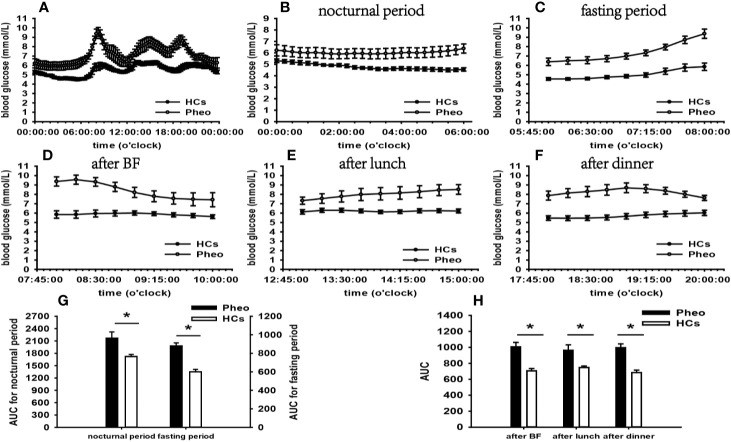
Comparison of the mean blood glucose level and AUC between the Pheo group and HCs. Mean blood glucose level: **(A)** at each time point during the day; **(B)** at nocturnal period; **(C)** at fasting period; **(D)** after BF; **(E)** after lunch; **(F)** after dinner. AUC: **(G)** at nocturnal and fasting periods; **(H)** at postprandial periods. * represents significant difference between Pheo and HCs. The data showed as mean ± SEM. AUC, area under the curve; Pheo, pheochromocytoma; HCs, healthy controls; BF, breakfast; SEM, standard error of mean.

**Figure 5:**
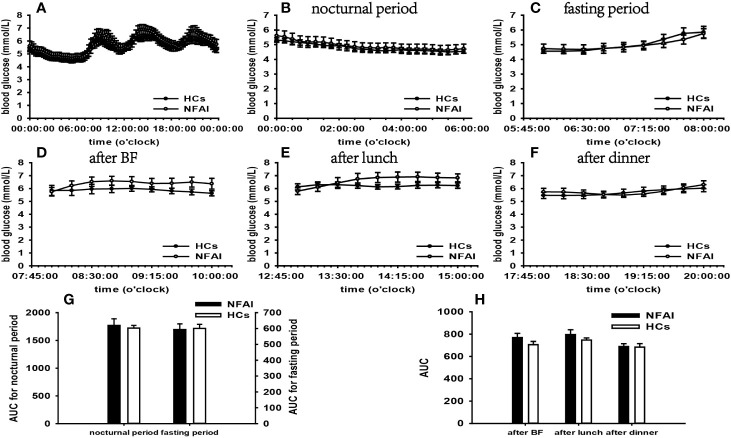
Comparison of the mean blood glucose level and AUC between the NFAI group and HCs. Mean blood glucose level: **(A)** at each time point during the day; **(B)** at nocturnal period; **(C)** at fasting period; **(D)** after BF; **(E)** after lunch; **(F)** after dinner. AUC: **(G)** at nocturnal and fasting periods; **(H)** at postprandial periods. The data showed as mean ± SEM. AUC, area under the curve; NFAI, nonfunctional adrenal incidentaloma; HCs, healthy controls; BF, breakfast; SEM, standard error of mean.

According to the results of specifically analyzed time periods, significantly different AUC was found at two postprandial periods in the CS group and the PA group compared with that in HCs, respectively; no significantly different AUC was identified at nocturnal and fasting periods in these two groups (**Figures 2**, **3**). A significant difference was reported in AUC at all five specifically analyzed time periods in the Pheo group (**Figure 4**). No significant AUC was observed in the NFAI group (**Figure 5**).

## Discussion

In the CS group, we observed significantly affected general blood glucose levels, more extensive within-day and day-to-day GV, and higher incidence of hyperglycemia compared with those in HCs according to FGMS-related parameters. However, general HbA1c level was not significantly higher than that in HCs. FGMS data revealed that glucose metabolism of the patients in our CS group was impaired by abnormal GCs secretion.

The seminal concept that GCs may perturb glycometabolism was first recognized by Houssay more than 60 years ago (22). Accumulating evidence has expanded this concept to date. Current knowledge indicates that GCs impair glycometabolism through various mechanisms. In the conditions of excessively secreted GCs, the gluconeogenesis in the liver is stimulated, and postprandial glucose uptake is suppressed in skeletal muscle and adipose tissue (23).

Furthermore, analysis of parameters representing IR revealed significant IR in the CS group than that in HCs, which indicated that the CS may influence glycometabolism through inducing IR. It is generally recognized that GCs function as a stimuli of lipolysis and proteolysis resulting in increased glycerol, fatty acids and amino acids, which in turn provide substrates for gluconeogenesis and induce IR (24), contributing to glycometabolism impairment. Significantly higher HOMA-β and FINS than those in HCs suggested better islet β-cell function and hyperinsulinemia, which contradicts the common knowledge that GCs could damage islet β-cell function (25). We inferred that the underlying mechanisms were compensatory β-cell enlargement and transient insulin over-secretion, which usually occurred at the early phase of IR in the patients with CS to counteract IR and maintain normal glucose levels (26). Impaired β-cell function presents when insufficient hyperinsulinemia fails to compensate for IR.

According to the results of specifically analyzed times periods, our data revealed that postprandial blood glucose levels were significantly affected, however, fasting and nocturnal blood glucose levels were generally unaffected in the CS group than in HC. The t-test result of FBG did not find significance compared with that in HCs.

There is growing evidence suggesting that approximately 64% of CS patients exhibit normal FBG, and impairment in glucose metabolism tends to occur at the postprandial phase (27, 28). A significant body of evidence highlights that impaired postprandial blood glucose levels are based on GCs’ contra-insulin effects in liver, skeletal muscle, and adipose tissue (23). Besides, Glycogen synthesis is attenuated in liver and stimulated in skeletal muscle in light of GCs’ opposite effect on glycogen synthase (29), which may play a role in normal FBG and elevated postprandial blood glucose (PBG). Furthermore, HOMA-β and FINS in the CS group were significantly higher than that in HCs, indicating that the compensatory hyperinsulinemia for IR should also be taken into account for the normal FBG (30).

A further important consideration is that cortisol secretion in healthy individuals exhibits circadian and pulsatile patterns, reaching a maximum in the morning and minimum at midnight, as well as transient over-secretion in response to stress (31). In CS patients, by contrast, normal cortisol secretion rhythm is lost. The atypical higher cortisol level at midnight leads to an increment of nocturnal blood glucose level in these patients. No indication of elevated nocturnal blood glucose emerged in our CS group. This observation contradicts previous findings and the possible cause underlying this phenomenon may be that the hyperinsulinemia counteracts GCs’ functions in raising blood glucose, contributing to the normal nocturnal blood glucose. Further investigations to explain this phenomenon are warranted.

In the PA group, significantly affected general blood glucose levels, more extensive within-day and day-to-day GV, and greater hyperglycemia incidence were identified by analysis of FGMS-related parameters. Furthermore, the general HbA1c level exceeded that in HCs. These results implied that patients in the PA group experienced glycometabolic deterioration.

The groundbreaking notion that glycometabolism impairment may be present in PA was put forward by Conn in the 1960s (32). Recent data document that the ALD is the major cause in this setting (33). A cluster of evidence indicates that ALD can activate enzymes responsible for gluconeogenesis (fructose-1,6-biphosphatase and phosphoenolpyruvate carboxykinase) and glucose delivery to the circulation (glucose-6-phosphatase), leading to amplified hepatic glucose production and release (34). The glucose transporter 4 and 2 gene expression is impaired by ALD, which results in defective glucose uptake in skeletal muscle, adipose tissue and liver (35).

A large array of observations highlight impaired insulin secretion and IR in patients with PA (36–38). However, there was no evidence that IR and impaired β-cell function occurred in our PA patients according to surrogate parameters analysis. Notable recent work indicates that patients with PA develop IR within a 10-year follow-up period despite the absence of IR at baseline (39). It is tempting to speculate that our patients were treated in their early stage of disease and have not progressed to IR.

Our findings revealed that significantly increased blood glucose levels occurred at postprandial periods not at nocturnal and fasting periods in the PA group. What is more, there was no significantly increased FBG compared with that of HCs. In combination of these results, we can conclude that PBG was significant affected in our PA group.

It has been demonstrated in animal experiments that hyperaldosteronism can induce elevated FBG depending on its stimulating effect on hepatic glucose release (40). Several clinical studies reveal higher FBG in PA compared with that of controls, whereas others yield discrepant outcomes (41, 42). Few studies have reported PBG levels in patients with PA. However, a series of evidence suggests that 2-h oral glucose tolerance test plasma glucose levels are higher in patients with PA than in controls (43, 44).

Hypokalemia is recognized as a possible player in impaired glucose metabolism of PA (45). A recent study indicates a negative correlation between potassium levels and 2-h oral glucose tolerance test blood glucose levels due to hypokalemia impairs insulin receptor function and insulin secretion facilitated by glucose (43, 46). The mean plasma potassium in our PA group was lower than the normal range. There is a propensity to interpret PBG changes as a result of hypokalemia. Another important insight is that prevailing cortisol cosecretion in PA may function in synergy with ALD, further exacerbating the glucose metabolism (40). Patients with hypercortisolism is susceptible to display increased PBG, as described above. A recent study indicates that higher PBG is more prevalent in PA with autonomous cortisol secretion than in PA without autonomous cortisol secretion (43). The cortisol rhythms in our PA patients were normal, and the mean cortisol level at 08:00 was within the normal range. However. the overnight 1-mg DST failed to be conducted. Therefore, the role of cortisol cosecretion in elevated PBG in our PA group remains uncertain.

In the Pheo group, FGMS monitoring data revealed significantly affected general blood glucose levels, more extensive within-day and day-to-day GV, and larger hyperglycemia PT than those in HCs. Significantly increased general HbA1c level compared with that of HCs was identified by a t-test. Besides, the analysis of blood glucose levels at different periods indicated that increased blood glucose levels occurred at nocturnal, fasting, and postprandial periods. The integration of these results suggested that glucose metabolism of our Pheo patients was significantly affected by the disease.

Convincing evidence indicates that nearly half of patients with Pheo are subjected to glycometabolism impairment (4). The underlying mechanisms is thought to be excessively secreted CA binding to adrenergic receptors. In the liver, gluconeogenesis and glycogenolysis are stimulated by β-adrenergic receptors (47). Glucose uptake is impeded by influences of CA occurring at the skeletal muscle level (48).

Significantly increased FBG and blood glucose levels at 06:00–08:00 than those in HCs suggested FBG was significantly influenced in patients of our Pheo group, which accords with previous findings (49, 50). In the fasting state, surging CA leads to incremental blood glucose levels due to amplified hepatic gluconeogenesis and glycogenolysis (51). Few attentions have been paid to nocturnal and postprandial blood glucose levels in these patients. By using FGMS, our study conducted dynamic blood glucose monitoring in patients with Pheo and analyzed blood glucose levels at multiple periods. Thus, we hypotheses that in addition to FBG, nocturnal and postprandial blood glucose levels can be significantly influenced by abnormal CA secretion according to our results. Further investigations are in prospect to prove this conclusion.

No significant differences in the indices of islet β-cell function and IR were observed in our study. There is an emerging consensus that impaired insulin secretion play a central role in the glycometabolism impairment in Pheo (52). By acting on α2 adrenergic receptors of islet β-cell, CA elicits inhibitory effects on insulin secretion (53). Recent notable research indicated that excessive CA induced or aggravated IR in Pheo patients (54). Lipolysis is facilitated *via* β-adrenergic mechanisms, increasing the level of free fatty acids, whose elevation not only provides the substrates for hepatic gluconeogenesis, but further exacerbates IR (49).

Reasons underlying the phenomenon of significantly impaired glucose metabolism and unaffected islet β-cell dysfunction remain unknown. One possibility is that other hormones produced by Pheo play a part in impaired glycometabolism. In addition to CA, some tumor-secreted peptides also disturb glucose metabolism. For example, the vasoactive intestinal peptide is akin to glucagon and leads to glycometabolic alterations by stimulating glycogenolysis and lipolysis (55). In addition, it is important to be aware that CA secretion presents as an uncontrollable manner with variable mount, type, and pattern in Pheo, such as low, high, or no CA secretion, intermittent or continuous secretory activity, and epinephrine, norepinephrine, or dopamine-dominate type (56). Different types of CA induce different effects, and epinephrine may be the dominated player in our Pheo patients. Evidence for the hypothesis comes from epinephrine’s preferential affinity to β-receptor, which stimulates gluconeogenesis (4). Furthermore, epinephrine is more potent than norepinephrine to increase blood glucose levels (57).

A further aspect requiring consideration is that increased PRA and secondary aldosteronism may play a part in the metabolic alterations in patients with Pheo since CA binding to β1-adrenoreceptor can directly stimulate renin release *via* the cAMP-PKA signaling pathway (58). However, the plasma PRA and ALD upright levels were within the normal range in our Pheo patients. A possible explanation of this phenomenon was that the stimulated renin release may be counteracted by increased renal perfusion pressure or elevated sodium concentration at the macula densa resulted from increased blood pressure (59).

In the NFAI group, greater fluctuations of general blood glucose levels were identified than that in HCs according to the t-test result of CV. More extensive day-to-day GV was demonstrated by significantly different ABPC compared with that of HCs. In addition, significantly decreased PT2 and TIR along with significantly increased TOR indicated poor glucose-target-rate. However, a higher hyperglycemia incidence failed to be concluded due to the non-significantly different PT1 and PT3. The analysis of blood glucose levels at different periods indicated that none of these periods was significantly affected by the disease.

Several studies have found a high prevalence of glucose metabolism aberrations in NFAI (60). Our study employed FGMS to observe dynamic glucose changes during the day, demonstrating that glucose metabolism impairment occurred at our NFAI patient and it was characterized by significantly affected general blood glucose levels, more extensive GV and poor glucose-target-rate. Due to the availability of overnight 1-mg DST data only in one patient, we cannot exclude the possibility of subclinical Cushing syndrome in other patients, though, whose ACTH, cortisol rhythm, midnight cortisol, and 24h urinary free cortisol data seem to suggest normal cortisol secretion. We recommended these patients endocrinological assessment and glucose monitoring in the further follow-up.

Based on our data, one could argue that adrenal diseases (CS, PA, Pheo, and NFAI), whose incidence is escalating in the general population have profoundly negative implications for glucose metabolism. The usage of FGMS in this study provides us consecutive and elaborate blood glucose changes during the surveillance. Compared with HbA1c, FBG, and 2-h oral glucose tolerance test blood glucose, FGMS achieves the observations of blood glucose levels at a certain period, enabling more accurate and reasonable assessment of blood glucose pattern at different periods. In addition, FGMS contributes to GV detection at general, within-day, and day-to-day levels, adding new evidence for glucose metabolism impairment. For example, patients with CS are susceptible to PBG changes, therefore, blood glucose management in these patients should focus on postprandial periods. Some CS patients may exhibit normal nocturnal blood glucose levels as a result of compensatory hyperinsulinemia as an adaption for IR. Our data also demonstrated significantly affected PBG in patients with PA. However, no significance was identified in FBG, which contradicts the previous studies. Nevertheless, we still recommended that attention should be paid to both fasting and postprandial periods for glucose management of patients with PA.

Furthermore, it is important to reiterate that there has been convincing proof of the strong relationship between impaired glucose metabolism, IR, and cardiovascular events. A consistent finding of many studies is the remarkable amelioration of blood glucose levels and IR after surgical treatment (61–64). Therefore, those patients with adrenal diseases presenting operative indications should receive surgery without delay. However, necessary glucose lowering treatment should recommend to reduce GV and maintain glycometabolism homeostasis for preoperative or poor risk patients.

Some limitations of our study should be noted. Firstly, FGMS achieved continuous blood glucose surveillance under the premise that the sensor was scanned by the reader within 8 h. As a consequence of poor compliance and other unavoidable factors, several blood glucose values were missed. Secondly, overnight 1-mg DST failed to be conducted in PA, Pheo, and NFAI groups to detect autonomous cortisol secretion. It is better to conduct a 1-mg DST in PA and Pheo groups to screen cortisol cosecretion, and in NFAI group to exclude subclinical Cushing syndrome. In addition, we failed to eliminated the effect of hypokalemia in this study. It is hormones which play a main part in glucose metabolism impairments in patients with adrenal diseases, and there have been notable studies reporting the persistence of insulin resistance after hypokalemia elimination by potassium supplement (65, 66). However, further investigations with large sample sizes after eliminating these limitations are in prospect to confirm our findings.

In conclusion, our study analyzed the characteristics of glycometabolism alterations in CS, PA, Pheo, and NFAI patients, combining with islet β-cell dysfunction and IR assessment, further elucidating the mechanisms contributing to these metabolic changes. We recommended that patients diagnosed with adrenal diseases should receive timely and appropriate treatment to avoid adverse cardiovascular events linked to impaired glycometabolism and IR.

## Data Availability Statement

All datasets presented in this study are included in the article/**Supplementary Material**.

## Ethics Statement

This study was reviewed and approved by the ethics committee in the First Hospital of Shanxi Medical University. The patients/participants provided their written informed consent to participate in this study.

## Author Contributions

MH, XC, CZ, LY, NY, PS, JY, FG, YR, JZ, and DL conducted the study, including recruit subjects and collect data. MH analyzed the data and interpreted the results. MH and XC drafted the manuscript. YL and YZ designed the study and revised the manuscript. All authors contributed to the article and approved the submitted version.

## Funding

This study is supported by National Nature Science Foundation of China (NO. 81670710, 81770776) and Research Project Supported by Shanxi Scholarship Council of China (2017-053, 2020-172).

## Conflict of Interest

The authors declare that the research was conducted in the absence of any commercial or financial relationships that could be construed as a potential conflict of interest.

## Abbreviations

ADs, adrenal disease groups; ABPC, area between 25–75 percentile curves; ALD, aldosterone; ARR, aldosterone to renin ratio; AUC, area under curves; BF, after breakfast; CA, catecholamine; CS, Cushing syndrome; CT, computed tomography; CV, coefficient of variance; DST, dexamethasone suppression test; FBG, fasting blood glucose; FGMS, flash glucose monitoring system; FINS, fast insulin; GCs, glucocorticoid; GV, glucose variability; G/I, fasting glucose to insulin ratio; HbA1c, glycosylated hemoglobin; HCs, healthy controls; IR, insulin resistance; MAGE, mean amplitude of glycemic excursions; MODD, mean of daily differences; NFAI, nonfunctional adrenal incidentaloma; PA, primary aldosteronism; PBG, postprandial blood glucose; Pheo, pheochromacytoma; PRA, plasma renin activity; PT, percentile time (PT1, <3.9mmol/L; PT2, 3.9–7.8mmol/L; PT3, >7.8mmol/L); QUICKI, quantitative insulin sensitivity check index; SDBG, standard deviation of blood glucose; TIR, time in range; TOR, time out of range; 24hMBG, 24-h mean blood glucose.
